# Infection Prevention and Control in Correctional Settings

**DOI:** 10.3201/eid3013.230705

**Published:** 2024-04

**Authors:** Newton E. Kendig, Sarah Bur, Justin Zaslavsky

**Affiliations:** George Washington University, Washington DC, USA (N.E. Kendig, J. Zaslavsky);; VitalCore Health Strategies, Topeka, Kansas, USA (S. Bur)

**Keywords:** infection prevention and control, jail, prison, detention, correctional, carceral, United States

## Abstract

Correctional facilities house millions of residents in communities throughout the United States. Such congregate settings are critical for national infection prevention and control (IPC) efforts. Carceral settings can be sites where infectious diseases are detected in patient populations who may not otherwise have access to health care services, and as highlighted by the COVID-19 pandemic, where outbreaks of infectious diseases may result in spread to residents, correctional staff, and the community at large. Correctional IPC, while sharing commonalities with IPC in other settings, is unique programmatically and operationally. In this article, we identify common challenges with correctional IPC program implementation and recommend action steps for advancing correctional IPC as a national public health priority.

During 2021, ≈7 million persons were admitted to US jails across 2,848 jurisdictions; at year end, 1.2 million persons were incarcerated in state and federal prisons (*1*,*2*). The population dynamics of jails and prisons are different. Jails largely house persons awaiting trial or with sentences <1 year. In 2021, the average detention time for jail residents was 33 days; weekly population turnover was 42%. In contrast, prisons largely house persons with sentences >1 year; residents spend an average of 2.7 years in state prisons (*3*). Consequently, healthcare services for jail residents are mostly focused on screening diagnostics upon admission and short-term interventions for critical health care needs, whereas the prison setting allows for ongoing diagnostic assessments, chronic disease management, and comprehensive discharge planning. The populations residing in US jails and prisons present unique challenges and strategic opportunities for infection prevention and control (IPC) efforts. We review these challenges and conclude with recommended action steps to advance correctional IPC as a national public health priority.

Infectious disease transmission in the carceral setting is amplified by crowded living conditions (*4*), poor ventilation, and the incarceration of vulnerable patient populations. Those populations include persons with low socioeconomic status, migrant populations, aging patients with chronic diseases, patients with substance use disorders and serious mental illness, and those living with bloodborne-pathogen infections (*5*–*10*). Widespread transmission of pathogens can occur easily because incarcerated persons interact frequently with other residents, correctional staff, volunteers, and visitors, and upon release they engage with family and community social contacts. Infectious disease outbreaks in the correctional setting are well documented and most notably include influenza, COVID-19, tuberculosis, hepatitis B virus infections, methicillin-resistant *Staphylococcus aureus* (MRSA) infections, varicella, ectoparasite infections, and foodborne illnesses (*11*–*21*). Those outbreaks can pose significant threats to the health of incarcerated residents and correctional staff and can markedly impact correctional operations.

The COVID-19 pandemic illustrated that infectious disease outbreaks in correctional settings can occur nationwide from a single emerging pathogen, with dire consequences. Incarcerated persons and correctional workers were highly vulnerable to SARS-CoV-2 infection (*12*–*15*,*22*). Among residents of state and federal prisons, the COVID-19 incidence rate was 3.3 times higher than that of the US general population and the COVID-19 mortality rate was 2.5 times the US general population (*23*). Containing COVID-19 was challenged by the frequent movement of incarcerated persons and correctional workers into, between, and out of correctional facilities. In 1 large urban jail, a COVID-19 outbreak contributed substantially to local community spread of SARS-CoV-2 (*12*). However, in the same jail, implementing IPC measures of enhanced sanitation, social distancing, universal masking, widespread diagnostic testing, medical isolation, and quarantine was highly effective in containing further outbreaks, highlighting the public health importance of correctional IPC efforts (*24*).

Beyond preventing and containing infectious disease outbreaks, effective correctional IPC programs can advance public health in multiple ways. Carceral settings can detect infectious diseases in patient populations who may not otherwise use health care services by implementing recommended screening strategies (*25*–*32*). Furthermore, residents diagnosed with communicable diseases such as HIV, syphilis, hepatitis C, and tuberculosis can be effectively treated while incarcerated (*33*–*38*). Finally, planning for residents transitioning to the community can include patient education on harm reduction strategies to prevent acquisition of infectious diseases, provision of HIV preexposure prophylaxis (PrEP) for patients with HIV risk factors, linkages to care for patients with communicable diseases who require treatment, and coordination with public health authorities when appropriate (*39*–*42*).

Despite its importance to public health, successful implementation of correctional IPC programs has been elusive in many jurisdictions. Amid competing priorities, correctional IPC programs must have strong engagement and support from facility leadership, dedicated personnel, and sufficiently allocated resources, including funding for IPC services in private-sector healthcare contracts. Such programs must also be highly adaptable and account for the diverse risk factors of residents; the wide range of housing situations, communal programming, and conditions of confinement; and the system-specific correctional policies and procedures that affect day-to-day operations.

Of note, US jails and prisons operate primarily as public safety institutions, not healthcare facilities. As a result, correctional systems may not prioritize support for IPC programs within their facilities. They also may fail to engage with relevant external stakeholders, such as public health authorities, the medical community, and academia, or may find those stakeholders unwilling to collaborate with them. The resulting isolation can lead to an inadequate exchange of useful surveillance and epidemiologic data on infectious diseases; a lack of technical support and consultation to inform IPC activities; and limited external research and evaluation to assess IPC programs and drive continuous quality improvement. Despite those challenges, partnerships between correctional systems and public health and academic medicine are feasible and have proven mutually beneficial (*43*–*45*).

An operational challenge for correctional IPC programs is identifying a qualified correctional infection preventionist (CIP) to manage the program with the oversight of an interdisciplinary IPC committee. An effective CIP must possess a rare combination of skills, including a thorough knowledge of highly technical and evolving IPC guidelines; the ability to translate this information into actionable policies and understandable educational messages for correctional leadership, staff, and patients; and the interpersonal skills to engage effectively with multiple internal and external stakeholders. The CIP is typically a registered nurse or licensed practical nurse who may or may not be assigned full-time to IPC duties. Their basic nursing education does not prepare them for the CIP role, and there is no formal healthcare professional training or certification for correctional IPC. Thus, CIPs must learn their discipline through mentorship, work experience, and general IPC educational offerings. This lack of formal recognition of the CIP profession is shortsighted, given its unique and important role in protecting public health.

Correctional IPC shares fundamental scientific principles with IPC in dedicated healthcare settings, but it is different programmatically and operationally (Table) (*46*). CIPs must manage a hand hygiene program that includes the use of alcohol-based hand sanitizer, which is a potential fire safety risk, and monitor facility sanitation that is often the responsibility of the residents, who may not be closely supervised nor adequately trained. CIPs must also develop facility-specific bloodborne pathogen exposure control plans, including procedures to minimize sharps exposures during security searches, as well as facility-specific tuberculosis control plans, including procedures for safely transporting patients with suspected tuberculosis in security vehicles. They coordinate the investigation and management of infectious-disease outbreaks, which are often complicated by dormitory housing, limited space for isolation of patients, the abrupt movement of residents within and between correctional facilities, and the reluctance of residents to disclose symptoms or behaviors because of stigma, medical co-pays, fear of disciplinary action, or fear of placement in medical isolation. CIPs must provide guidance on housing and disinfection to prevent the transmission of *Clostridioides difficile* and *Candida auris* from recently hospitalized residents. Their promotion of proven harm-reduction strategies, such as condom distribution, may be discouraged or prohibited. In some jurisdictions, CIPs may also manage occupational health programs such as tuberculosis screening, vaccinations, and personal protection equipment that may be complicated by challenging labor–management relations.

**Table:** Correctional infection prevention and control challenges, United States*

Infection prevention and control domains	Infection prevention and control challenges
Outbreak risk and management	Correctional facilities are high-risk congregate settings for infectious disease outbreaks, such as influenza, COVID-19, tuberculosis, norovirus, varicella, and ectoparasites.
	Incarcerated residents may be more vulnerable to communicable diseases, including vaccine-preventable illnesses.
	Outbreak management is complicated by limited isolation capacity and the frequent movement of residents within and between correctional facilities.
Admission screening	Implementing evidence-based screening recommendations for infectious diseases may be complicated by the high volume of new admissions, brief periods of detention, health literacy barriers to patient history taking, cursory physical examinations, and lack of rapid testing capabilities.
Social distancing	Overcrowding and dormitory housing of incarcerated resident populations facilitate disease transmission and limit the feasibility of social distancing.
Hand hygiene	Resident access to liquid or foam soap, running water, and disposable paper towels may be limited.
	Access to flammable alcohol-based hand sanitizer may be restricted or prohibited due to fire safety concerns and risk of consumption by residents.
Sanitation and laundry	Cleaning of housing units is routinely performed by the residents themselves who may not have adequate training or supplies.
	Cleaning of common areas and shared equipment may be inadequate, e.g., intake processing areas, programming spaces, telephones, computers, recreational equipment, and security restraints.
	Residents commonly handwash and air dry their clothing which provides inadequate disinfection.
Bloodborne pathogen exposures	Resident access to bleach is routinely prohibited.
	Correctional staff and residents may be unexpectedly exposed to blood and other potentially infectious materials through physical assaults and altercations.
	Correctional staff may be exposed to sharps, such as tattoo needles and homemade shanks, during body searches of residents.
	Residents may be exposed to bloodborne pathogens from sharing needles for tattooing and injection drug use and from having sexual exposures without barrier protections.
Harm reduction	Harm reduction strategies to reduce infectious disease transmission, such as condom distribution, certified tattooing for residents, and needle exchange programs, are largely prohibited for security or regulatory reasons.
Housing challenges	Airborne isolation units and medical isolation single cell capacity may be nonexistent or very limited in number.
	Quarantining residents may be difficult due to overcrowding and lack of housing options.
	The conditions of confinement associated with medical isolation, quarantine, and facility-wide lockdowns can negatively impact the mental health of residents and limits their access to correctional programs.
	Long-term housing options, that are not socially isolating, may be unavailable for residents with healthcare-acquired infections, such as *C. auris.*
Resident transport	Security vehicles are not configured to prevent the transmission of infectious diseases.
	Disinfection of security vehicles may be inadequate due to operational constraints and lack of evidence-based protocols.
Correctional operational factors	The movement of residents and correctional staff between correctional facilities, courts, and the community is highly dynamic and difficult to minimize.
	Frequent personnel shortages of correctional staff negatively impact the implementation of infection prevention and control policies and procedures.
	The carceral environment may discourage symptomatic residents with contagious diseases from seeking medical attention due to stigma, medical co-pays, fear of disciplinary action, or fear of placement in medical isolation.
	Implementing occupational health recommendations for correctional staff, such as guidance on immunizations and personal protective equipment, may be complicated by challenging labor-management relations.
Discharge planning	Discharge planning of residents to prevent further transmission of communicable diseases may be complicated by a lack of continuity for antimicrobial treatments, insufficient medical insurance coverage, inconsistent access to harm reduction strategies, and difficulties securing substance use disorder treatments, safe housing, and psychosocial support.

*Adapted from (*46*).

CIPs must also provide IPC guidance for highly unusual or complex infectious disease scenarios unique to carceral settings. Examples include preventing and managing botulism cases from residents drinking illicitly made alcoholic beverages (*47*), preventing bloodborne pathogen transmission from unregulated tattooing, and managing foodborne outbreaks that result from residents sequestering and consuming inadequately stored perishable food. In addition, CIPs may be tasked with managing uncommon outbreaks of highly communicable diseases such as measles, mumps, and varicella, which have particularly affected immigrant detention facilities (*48*,*49*). Lastly, CIPs must develop and implement facility response plans for rapidly evolving emerging pathogens when initial public health guidance is limited, as was required with the COVID-19 pandemic and the recent mpox outbreak (*50*).

To be fully effective, CIPs must be supported by correctional staff across departments. The facility’s leadership must convey the importance of IPC policies to all correctional staff and empower the CIP to advise department heads who supervise correctional programs for security, food services, recreation, and sanitation. The facility’s clinical authority must provide sound medical guidance to the CIP and engage actively in IPC administrative meetings. In addition, the facility’s healthcare administrator must support IPC policy issuance and assign IPC tasks to appropriate healthcare team members. Last, correctional management officials and healthcare team members are critically dependent on the strong support of public health authorities. Timely promulgation of corrections-focused and population health–based IPC guidance enables the effective response to emerging pathogens and adoption of best practices.

Implementing more effective IPC programs in US jails and prisons will require a concerted investment from correctional officials, policy makers, public health authorities, health care professional organizations, academia, and other key stakeholders. The input of front-line correctional staff and persons who have experienced incarceration should inform these efforts.

Action steps should include the following:

1. Train and deploy a capable IPC workforce
  - Provide sufficient and adequately resourced personnel to manage IPC programs in US correctional facilities
  - Recognize the CIP as a unique IPC professional discipline that is supported by a national online training curriculum with certification requirements
  - Develop correctional-specific tools and checklists to drive excellence and standardize IPC practice
2. Improve surveillance
  - Improve infectious disease surveillance within correctional facilities by standardizing reporting requirements across systems, linking infectious disease reporting to a history of confinement in correctional settings, and evaluating innovative strategies (such as correctional facility wastewater surveillance) to detect pathogens
3. Augment evidence-based guidance
  - Augment evidence-based IPC guidance developed specifically for correctional facilities by the CDC and other national public health authorities
4. Strengthen external stakeholder engagement
  - Allocate federal funding to state and local health departments that encourages strong partnerships with correctional facilities to support key IPC activities such as vaccination programs, screening, surveillance and treatment of infectious diseases, outbreak management, and discharge planning coordination with community partners
  - Strengthen the IPC programmatic requirements of national detention standards that are promulgated by the federal government and standards of national organizations that provide healthcare accreditation to US jails and prisons
  - Develop and fund a national research agenda to evaluate the implementation of IPC recommendations in the correctional setting to identify best practices

Establishing successful correctional IPC programs in thousands of US jails and prisons is no easy task. Key stakeholders must act now at the local, state, and federal levels. The lessons learned from COVID-19 must inform current correctional IPC practices as well as future pandemic planning. Such investments are vital to the well-being of incarcerated residents, to the safety of correctional workers, and to the public health of our communities.
